# The Treatment of Hemorrhoids

**Published:** 1886-03-20

**Authors:** F. H. Rorick

**Affiliations:** Wauseon, Ohio


### THE TREATMENT OF HEMORRHOIDS.

By F. H. Rorick, M. D., Wauseon, Ohio.

I read with much interest Dr. Chas. B. Kelsey's rules for the treatment of hemorrhoids by injections of carbolic acid, in the Age of Nov. 25th, because of their eminent source.

During the last few years I have given the treatment of diseases of the rectum special attention and as some of my conclusions are at variance with those of Dr. Kelsey's, it might not be inopportune to give some of the results of my experience for the consideration of your readers.

The treatment of hemorrhoids by injections of carbolic acid is an important subject, for, as Dr. K. very aptly says, on the whole it seems to be the best method of treatment yet devised," and the general practitioner shows little disposition to employ the knife or ligature; therefore its adoption seems to be the only means whereby the legitimate practitioner can protect himself and drive from his field unreliable itinerants, who cure hemorrhoids by injection and claim to find conditions that do not exist.

There are few surgical procedures that are as easy of execution, or attended with as little danger to the patient, as the injection of hemorrhoids with a proper compound containing carbolic acid as the curative agent, and an impression otherwise given or formed is pernicious in its results. The selection of the purest medicines obtainable is a timely precaution whenever we bring them into requisition for the cure of disease. The same pathological conditions exist in a preponderance of all cases of internal hemorrhoids, and a compound of one strength will cure all; therefore it is useless to impose upon the beginner the embarrassment of guessing what strength solution is applicable in a given case, which in time, would of course result in experience. I have found a very weak solution (when injected in sufficient quantity to cure) as apt to produce a slough as a very strong one.

A slough is not necessary to the cure of any hemorrhoid except the arterial variety, which involves the anal membrane and is held in the grasp of the external sphincter. A 30 to 40 per cent. solution will very rarely produce a slough if properly deposited. I have injected several hundred times a compound containing carbolic acid 30 to 40 per cent., normal liquid ergot (P., D. & Co.) 15 per cent., and the balance water in double the quantity of glycerin to make the 100 per cent., and have never met with an accident except when I have inadvertently deposited the compound in the rectal wall. Even when this is done, the usual result is nothing more than a painful inflammation which passes off in a few days.

About two years ago I left my office in charge of a physician during a few days' absence. Upon my return I was summoned to the bedside of a gentleman whom the doctor had treated for hemorrhoids. He was suffering greatly from pain in the rectum and prostatic irritation. I learned from the doctor and examination of the case, that he had injected into the anterior wall of the rectum 1 drachm of a 40 per cent. compound. I anticipated trouble; however, in due time all turned out well, and much to my surprise, *no slough formed.*

This compound is not a perfect solution. The ingredients separate on standing, and it becomes turbid when subjected to a low temperature, but it is a clear solution at 80° or 90°, and should be warmed, as should also the hypodermic syringe. before using in cold weather. Three to 8 drops may be injected, according to size of tumor.

The manner in which the injection is made is of more importance than the compound used, for a cure will result from the injection of absolute alcohol as well as of the many vegetable and mineral astringents. In this connection I will relate a rather novel cure. Last summer, while in St. Louis, Mo., I was invited by a physician in charge of a private infirmary, to witness some operations. Among the rest was a case of hemorrhoids. Preparations were made to remove a large protruding tumor with the actual cautery. While administering the anæsthetic the subject of cure by injection came up, and the doctor concluded to change his course and inject absolute alcohol. After this was accomplished he thought to make assurance doubly sure by yet employing the cautery. No sooner did the iron penetrate the tumor than the alcohol ignited, which resulted in a total destruction of the hemorrhoid as well as the hair and some cuticle surrounding the anus, and it seems needless to state that the treatment was barbaric, and the cure was absolute.

Hemorrhoids should never be forced through the anus for injection, or injected while protruding, for the reasons that the straining effort and action of the sphincters over-distends the tumors, which makes it extremely difficult to properly deposit the compound and greatly enhances the liability to the formation of a slough. It imposes unnecessary after-suffering upon the patient, and small sacs located high up must of necessity escape detection and treatment, for they cannot be forced to view, neither will a digital examination reveal their presence. Therefore, when sufficient time elapses for the undiscovered tumors to so increase in size as to make their presence known, a return cf the disease is announced. Thus the reputation of the treatment suffers unjustly.

All injections should be made within the rectum, and this cannot be satisfactorily accomplished with a speculum that allows the membranes to enter throughout its entire length, which is the case with the bi-valve, tri-valve, and fenestrated varieties. With a view of accomplishing the end desired, I have improvised a cylindrical slide speculum, having an open inner end and provided with a plug which extends beyond the opening, to facilitate introduction. After the instrument is introduced the plug is removed and the slide, which extends the entire length of one side of cylinder, can be slowly withdrawn and the membrane carefully scrutinized as it enters the speculum over the end of the slide. It can be stopped wherever desired and nothing enters the speculum to obstruct the external view to the point under observation or treatment. The slot extending to the end obviates the objection to the Hilton and O'Neil slide speculum, for it does away with the sharp shoulder at the inner end of the slot which catches the membranes and produces no small amount of pain upon their withdrawal.

As the tumor appears over the slide introduce the point of the needle into its most dependent part and deposit the compound immediately underneath the mucous membrane. Do not aim to inject the body of the tumor. A large hemorrhoid may be injected in this manner in three or four different places at one sitting. The syringe for this purpose should work easily and the needle should be of platinum or steel, well sharpened and set in a canula of small calibre, three and one-half inches in length; side handles are not necessary.

By following these rules, no accident of moment need be apprehended, even at the hands of a novice. In my experience, very rarely a patient loses a day from his usual avocation as a result of treatment, and the cure is certain. In view of the fact that we can now successfully treat not only hemorrhoids, but prolapsus and even fistula by injections of proper remedies, the profession at large should arouse to a sense of its duty and make it no longer a truthful saying "that the surgery of the rectum is a sort of land of the Cimmerians, where quacks alone can breathe, and where humbug darkens the air."—*Medical Age.*

